# Retraction: Concomitant Targeting of EGF Receptor, TGF-beta and Src Points to a Novel Therapeutic Approach in Pancreatic Cancer

**DOI:** 10.1371/journal.pone.0233436

**Published:** 2020-05-13

**Authors:** The PLOS ONE Editors

Following the publication of this article [1], concerns have been raised regarding Figs 1, 4 and S3:

- The HA-tag panels representing ASPC-1 and T3M4 in Fig 1A appear similar.
- Figs 1A and 4A: ERK lanes 1–3 in Fig 1A (ASPC-1) appear similar to the ERK lanes 2–4 in Fig 4A (ASPC-1) when stretched horizontally.
- Figs 1A and S3: EGFR lanes 1–2 in Fig 1A (ASPC-1) appear similar to EGFR lanes 5–6 in S3 Fig (T3M4) when flipped horizontally.
- The EGFR lanes 1–4 (ASPC-1) and the pEGFR lanes 1–4 (ASPC-1) of Fig 4A appear similar when stretched vertically.
- Figs 4A and S3: HER2 lanes 1–4 in Fig 4A (T3M4) appear similar to HER2 lanes 1–4 (T3M4) in S3 Fig.
- Figs 4B and S3: ERK lanes 1–4 in Fig 4B (T3M4) appear similar to ERK lanes 1–4 (T3M4) in S3 Fig.

Original data for the panels of concern are no longer available. Replication data have been provided in support of the results presented in these panels. However, the concerns about western blot data reporting in the original figures remain unresolved.

In light of the concerns affecting multiple figure panels that question the integrity of the results, the *PLOS ONE* Editors retract this article.

SD and MM did not respond. MK did not agree with the retraction.
